# N6-Methyladenosine-Related lncRNAs as potential biomarkers for predicting prognoses and immune responses in patients with cervical cancer

**DOI:** 10.1186/s12863-022-01024-2

**Published:** 2022-01-18

**Authors:** He Zhang, Weimin Kong, Xiaoling Zhao, Chao Han, Tingting Liu, Jing Li, Dan Song

**Affiliations:** Department of Gynecological Oncology, Beijing Obstetrics and Gynecology Hospital, Beijing Maternal and Child Health Care Hospital, Capital Medical University, Beijing, 100006 China

**Keywords:** m^6^A, Tumour microenvironment, Stroma, Immunotherapy, Cervical cancer

## Abstract

**Background:**

Several recent studies have confirmed epigenetic regulation of the immune response. However, the potential role of RNA N6-methyladenosine (m^6^A) modifications in cervical cancer and tumour microenvironment (TME) cell infiltration remain unclear.

**Results:**

We evaluated and analysed m^6^A modification patterns in 307 cervical cancer samples from The Cancer Genome Atlas (TCGA) dataset based on 13 m^6^A regulators. Pearson correlation analysis was used to identify lncRNAs associated with m^6^A, followed by univariate Cox regression analysis to screen their prognostic role in cervical cancer patients. We also correlated TME cell infiltration characteristics with modification patterns. We screened six m^6^A-associated lncRNAs as prognostic lncRNAs and established the prognostic profile of m^6^A-associated lncRNAs by least absolute shrinkage and choice of operator (LASSO) Cox regression. The corresponding risk scores of the patients were derived based on their prognostic features, and the correlation between this feature model and disease prognosis was analysed. The prognostic model constructed based on the TCGA-CESC (The Cancer Genome Cervical squamous cell carcinoma and endocervical adenocarcinoma) dataset showed strong prognostic power in the stratified analysis and was confirmed as an independent prognostic indicator for predicting the overall survival of patients with CESC. Enrichment analysis showed that biological processes, pathways, and markers associated with malignancy were more common in the high-risk subgroup. Risk scores were strongly correlated with the tumour grade. ECM receptor interactions and pathways in cancer were enriched in Cluster 2, while oxidative phosphorylation and other biological processes were enriched in Cluster 1. The expression of immune checkpoint molecules, including programmed death 1 (PD-1) and programmed death ligand 1 (PD-L1), was significantly increased in the high-risk subgroup, suggesting that this prognostic model could be a predictor of immunotherapy.

**Conclusions:**

This study reveals that m^6^A modifications play an integral role in the diversity and complexity of TME formation. Assessing the m^6^A modification patterns of individual tumours will help improve our understanding of TME infiltration characteristics and thus guide immunotherapy more effectively. We also developed an independent prognostic model based on m^6^A-associated lncRNAs as a predictor of overall survival, which can also be used as a predictor of immunotherapy.

**Supplementary Information:**

The online version contains supplementary material available at 10.1186/s12863-022-01024-2.

## Background

In all organisms, genetic information flows from DNA to RNA and then to proteins. As the third layer of epigenetics, RNA plays a crucial role, not only in transmitting genetic information from DNA to proteins but also in regulating various biological processes. More than 150 RNA modifications have been identified, including 5-methylcytosine (M^5^C), N6-methyladenosine (M^6^A), and N1-methyladenosine (M^1^A), among others [1]. As the predominant and most abundant form of internal modification in eukaryotic cells, m^6^A is methylation occurring at the adenosine N6 position with an abundance of 0.1–0.4% among the total adenosine residues and it is widely present in mRNA, lncRNA and miRNA [2]. N6-methyladenosine is mainly present in two sequences, -G-m^6^A-C- (70%) and -A-m^6^A-C- (30%) [3], and it is enriched near the stop codon, 3' untranslated region (UTR) and in long internal exons [4, 5]. Three major classes of proteins are involved in m^6^A modification: the first is the methyltransferases responsible for the modification, the second is demethylases, and the third is effector proteins. m^6^A methylation is formed by methyltransferases such as RBM15, ZC3H13, METTL3, and METTL14, while the removal process is mediated by demethylases such as FTO and ALKBH5 [6]. In addition, a specific set of RNA-binding proteins, such as YTHDFs, IGF2BPs, and THDC1/2, can recognize m^6^A motifs and thus affect the function of m^6^A [7, 8]. An in-depth understanding of these regulatory factors will help to reveal the role and mechanism of m^6^A modifications in posttranscriptional regulation. It has been reported that m^6^A regulators play critical roles in a variety of biological functions in vivo. An increasing number of studies have shown that aberrant expression and genetic alterations of m^6^A regulators are associated with a variety of biological processes, including dysregulated cell death and proliferation, developmental defects, malignant tumour progression, impaired self-renewal capacity, and abnormal immune regulation [9–11].

Using the immune system to fight cancer has become an effective treatment option, and immunotherapy represented by immune checkpoint blockade (ICB, PD-1/L1, and CTLA-4) has shown impressive clinical efficacy in several cancer types [12, 13]. Unfortunately, the clinical benefit for most patients remains relatively small and far from what is needed to satisfy clinicians. Traditionally, we have considered tumour progression to be a multistep process involving only genetic and epigenetic variation in tumour cells [14]. However, numerous studies have shown that the microenvironment in which tumour cells grow and survive also plays a crucial role in tumour progression. The tumour microenvironment (TME) contains not only cancer cells but also stromal cells (e.g., resident fibroblasts, cancer-associated fibroblasts (CAFs)) and macrophages, as well as distantly recruited cells such as infiltrating immune cells (myeloids and lymphocytes), bone marrow-derived cells (BMDCs), and secreted factors such as cytokines, chemokines, growth factors, and neointima [15]. With the increasing understanding of the diversity and complexity of the tumour microenvironment, there is increasing evidence that the tumour microenvironment plays an important role in tumour progression and immune escape and has an impact on the immunotherapeutic response [16]. Predicting the ICB response based on the characteristics of TME cell infiltration is a critical step to improve the success of existing ICBS and to develop new immunotherapeutic strategies [17]. Thus, by analysing the heterogeneity and complexity of the TME landscape, it is possible to identify distinct tumour immunophenotypes, and the ability to guide and predict immunotherapeutic responses will be improved. Additionally, we aimed to reveal new relevant biomarkers and demonstrate the effectiveness of these markers in identifying patient responses to immunotherapy, with the goal of finding new relevant therapeutic targets.

In recent years, several studies have proposed a correlation between TME immune cell infiltration and m^6^A modifications [18]. Some evidence has demonstrated that m^6^A regulates transcriptional and protein expression through splicing, translation, degradation, and export, thereby mediating the biological processes of cancer cells and/or stromal cells and characterizing the TME [19]. The TME plays a critical role in the complex regulatory network of m^6^A modifications and it subsequently affects tumorigenesis, tumor progression, and the tumor therapeutic response [20]. Wang et al. showed that RNA methyltransferase METTL3-mediated m^6^A methylation promotes dendritic cell (DC) activation and function. m^6^A translation of METTL3-mediated CD40, CD80, and TLR4 signalling junction TIRAP transcripts is enhanced in DCs to stimulate T cell activation and enhance TLR4/NF-κB signalling-induced cytokine production [8]. Research by Jiang et al. showed that highly expressed TLR4 activated the NF-κB pathway, upregulated ALKBH5 expression, and increased m^6^A levels and NANOG expression, all contributing to ovarian carcinogenesis [21]. Chen et al. showed that m^6^A methylation of RNA and HIF-1α/2α-dependent AlkB homologue 5 (ALKBH5) participate in the regulation of HIFs and SOX2 in endometrial carcinoma. Hypoxia induces an endometrial cancer stem-like cell phenotype via HIF-dependent demethylation of SOX2 Mrna [22]. However, studies of the relationship between m^6^A and TMB interactions in cervical cancer have rarely been reported.

In general, basic research may be limited to only one or two M^6^A regulators and cell types. However, it is well known that antitumour effects are characterized by the interaction and high synergy of numerous tumour suppressors. Therefore, a comprehensive understanding of multiple m^6^A regulator-mediated TME cell infiltration patterns will help deepen our understanding of TME immune regulation [23]. In this study, we integrated genomic information from 307 cervical cancer specimens, performed a comprehensive evaluation of M^6^A modification patterns, and correlated M^6^A modification patterns with TME cell infiltration characteristics. We established an m^6^A-related lncRNA-based scoring system to quantify the m^6^A modification patterns of individual patients.

## Methods

### Cervical cancer dataset source and preprocessing

**Fig. 1:**
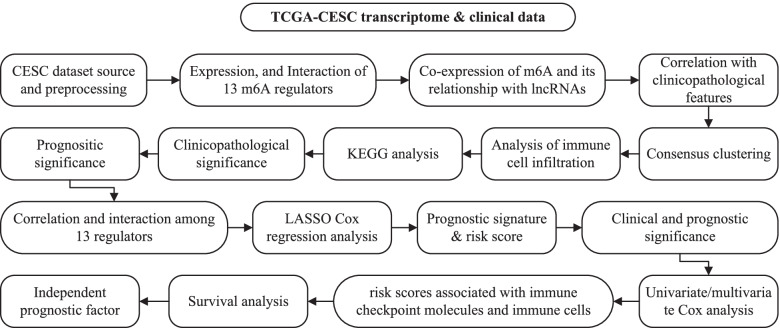
Flow chart of the development and validation of an N6-methylandenosine-related lncRNA-based prognostic signature for CESC

The workflow of our study is shown in Fig. 1. Public gene expression data and full clinical annotation were searched in the TCGA database. Patients without survival information were removed from the analysis. In this study, TCGA-CESC was collected for further analysis, which included a total of 307 tissue samples from patients with cervical cancer, as well as 3 normal tissue samples. RNA sequencing data (FPKM value) of gene expression were downloaded from the Genomic Data Commons (GDC, https://portal.gdc.cancer.gov/) [24]. Then, the FPKM values were transformed into transcripts per kilobase million (TPM) values. Coexpression analysis of m^6^A-associated genes and lncRNA-associated genes was performed using the "limma" package. Gene coexpression network relationship graphs were constructed using the "igraph" package.

### Unsupervised clustering for 13 m^6^A regulators

A total of 13 regulators were extracted from TCGA datasets to identify different m^6^A modification patterns mediated by m^6^A regulators. These 13 m^6^A regulators included 6 writers (METTL3, METTL14, RBM15, WTAP, KIAA1429, and ZC3H13), 2 erasers (ALKBH5, FTO), and 5 readers (YTHDC1, YTHDC2, YTHDF1, YTHDF2, and HNRNPC). Unsupervised clustering analysis was applied to identify distinct m^6^A modification patterns based on the expression of 6 m^6^A regulators and to classify patients for further analysis. The number of clusters and their stability were determined by the consensus clustering algorithm. We used the R package “ConsensuClusterPlus” to perform the above steps, and 1000 repetitions were conducted to guarantee the stability of the classification [25].

### Estimation of TME cell infiltration and functional annotation

We used the GSEA (gene-set enrichment analysis) algorithm to quantify the relative abundance of each cell infiltration in the CESC TME, including activated CD8 T cells, activated dendritic cells, macrophages, natural killer T cells, regulatory T cells, and so on. GSEA was performed using GSEA software, and gene sets of “c2.cp.kegg.v7.2.symbols” were downloaded from the MSigDB database (http://software.broadinstitute.org/gsea/msigdb) for running GSEA. Among them, KEGG has been widely used in biological big data analysis [26–28]. The enrichment scores calculated by GSEA were utilized to represent the relative abundance of each TME infiltrating cell in each sample. We regarded the pathways with |NES|> 1 and NOM p-val < 0.05 as significantly enriched pathways.

### Construction of the Prognostic Signature

The m^6^A methylation regulators were included in the least absolute shrinkage and selection operator (LASSO) Cox regression model. Prognostic features and correlation models were constructed, their correlation coefficients were calculated, and the expression of each gene was multiplied by its coefficient to calculate the sum of risk scores for each patient. The sensitivity and specificity of the prognostic signature were assessed by receiver operating characteristic (ROC) curves and the area under the ROC curves (AUC).

### Statistical analysis

The survival curves for the prognostic analysis were generated via the Kaplan–Meier method, and log-rank tests were utilized to identify the significance of the differences. We adopted a univariate Cox regression model to calculate the hazard ratios (HRs) for m^6^A regulators and m^6^A phenotype-related genes. The independent prognostic factors were ascertained through a multivariable Cox regression model. Patients with detailed clinical data were eligible for final multivariate prognostic analysis. The forest plot R package was employed to visualize the results of the multivariate prognostic analysis for the m^6^Ascore in the TCGA-CESC cohort. The specificity and sensitivity of the m^6^Ascore were assessed through the ROC curve, and the AUC was quantified using the “timeROC” R package. All statistical P values were two sided, with *p* < 0.05 defined as statistically significant. All data processing was conducted in R 4.0.4 software.

## Results

### Expression, Correlation, and Interaction of M^6^A methylation regulators in cervical cancer

**Fig. 2:**
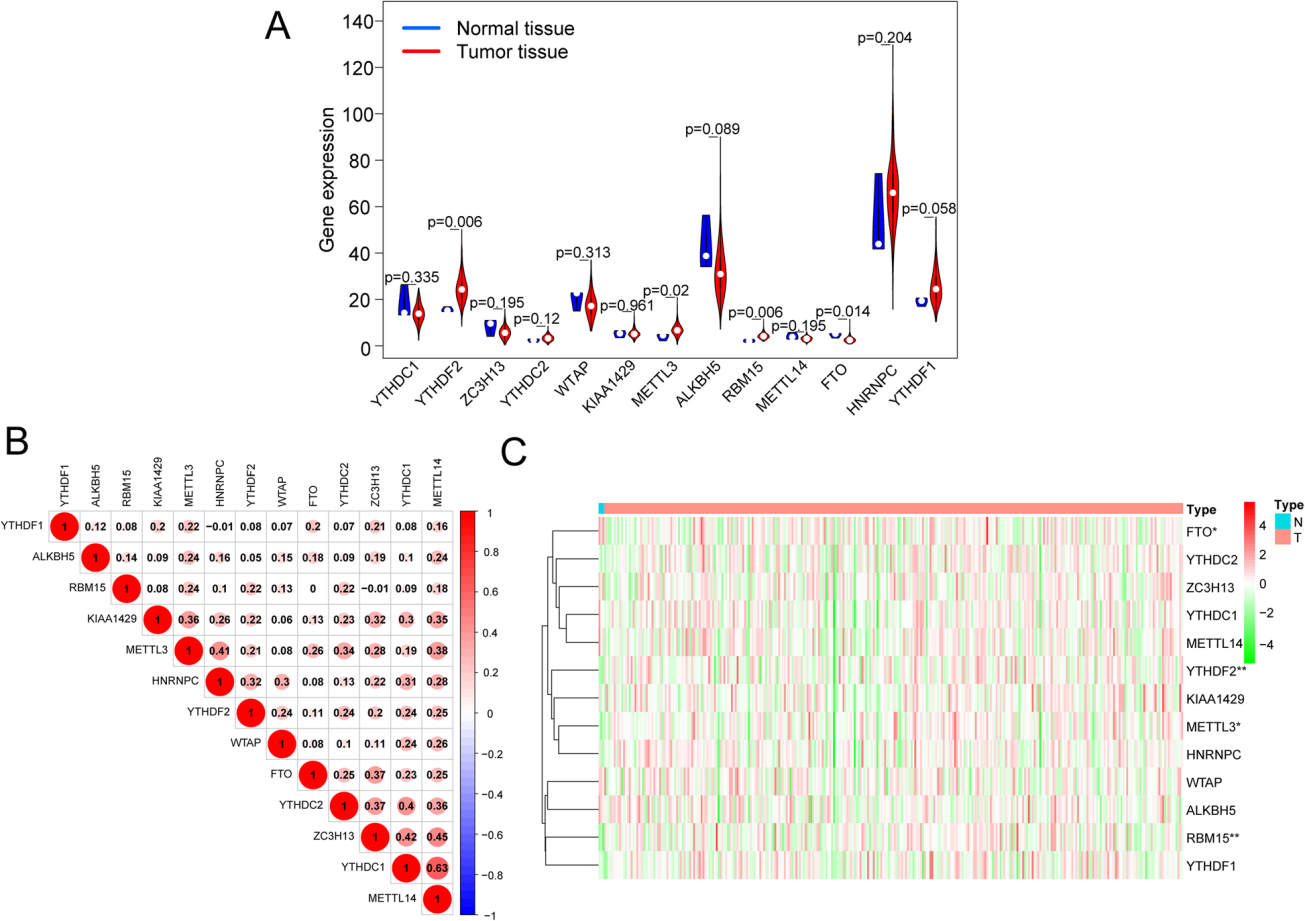
The expression of 13 m^6^A RNA methylation regulators in the TCGA-CESC cohort. **A** The violin plot shows the significantly differentially expressed m^6^A RNA methylation regulators between CESC tissues and normal tissues. **B** The correlations among m^6^A RNA methylation regulators were analysed by Pearson correlation. **C** Heatmap of m^6^A RNA methylation regulators between CESC tissues and normal tissues

The mRNA expression levels of m^6^A RNA methylation regulators were analysed using the transcriptome data in FPKM format. The expression levels of different m^6^A genes in normal and tumour tissues were observed and analysed differently by heatmaps with the R package "pheatmap" (Fig. 2C), and the expression levels of 13 regulators in CESC and normal tissues were shown in correlation plots of the R package "corrplot" (Fig. 2B) and the violin plot of "vioplot" (Fig. 2A). The results showed that the regulators were positively correlated with each other, including a significant positive correlation between YTHDC1 and METTL14, with a correlation coefficient of 0.63. The mRNA expression levels of three regulators (RBM15, METTL3, and YTHDF2) were significantly increased, and FTO was decreased in CESC compared with normal tissues. No significant difference was found for the other nine regulators.

### Coexpression of m^6^A and its relationship with lncRNAs and the search for prognosis-related lncRNAs

**Fig. 3:**
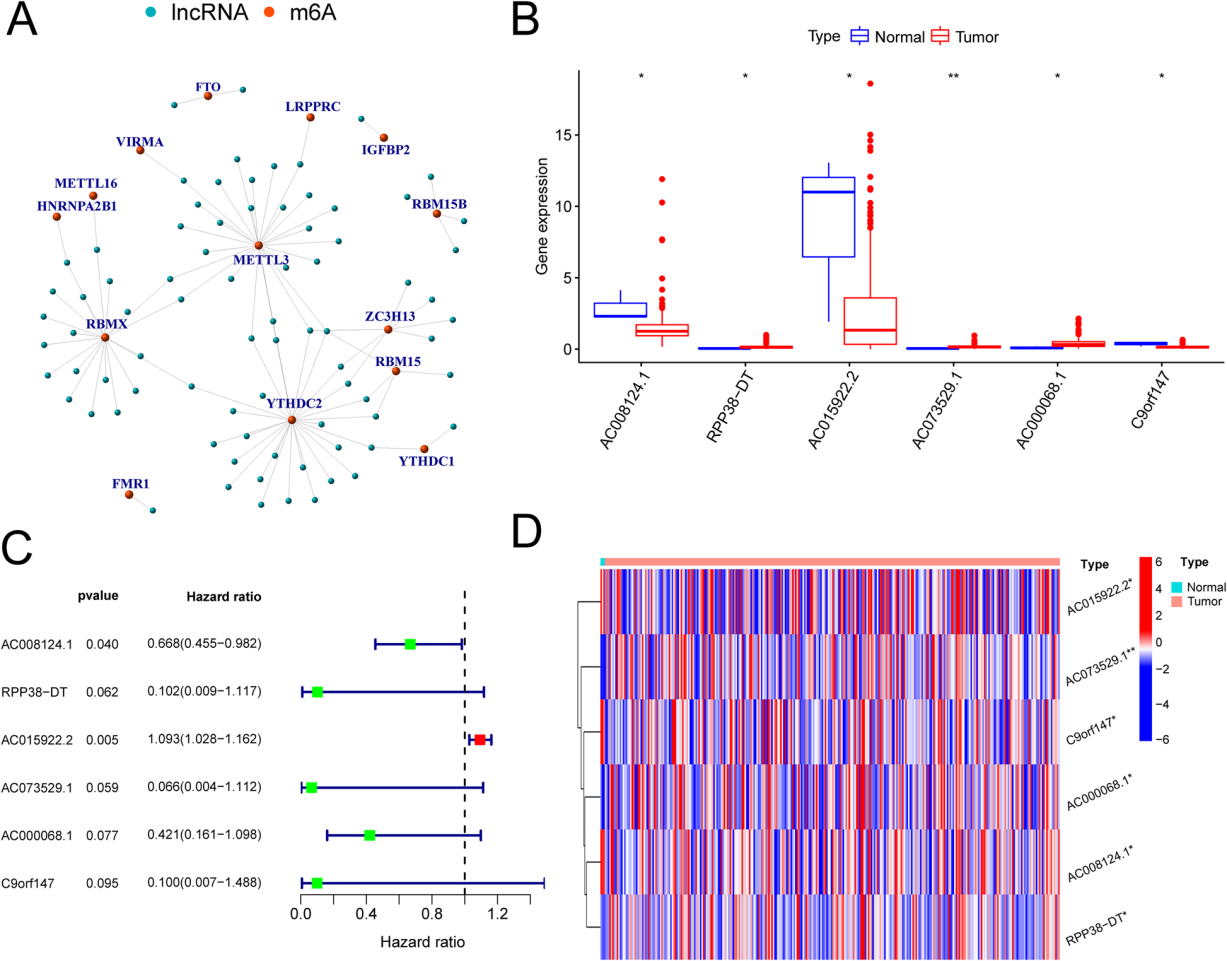
**A** Coexpression of m^6^A and its relationship with lncRNAs. **B** Expression of target lncRNAs in tumour samples and normal samples. **C** Forest plot of lncRNA expression by one-way Cox analysis, where red represents high-risk lncRNAs and green represents low-risk lncRNAs (*p* < 0.1). **D** Heatmap of lncRNA expression in normal and tumour samples. Red represents upregulated expression, and blue represents downregulated expression

Although the functions of most lncRNAs are currently not fully known, synergistic regulatory relationships or functional correlations between lncRNAs and mRNAs have been suggested to exist. Therefore, by constructing a coexpression network (Fig. 3A) of lncRNAs and mRNAs, we can predict the possible role of lncRNAs in cervical cancer. The m^6^A-related lncRNAs were identified by coexpression analysis with the R package "limma". m^6^A and lncRNA coexpression relationships were plotted with the R package "igraph". Six prognosis-associated lncRNAs, AC008124.1 (*p* = 0.04, HR = 0.668), AC015922.2 (*p* = 0.005, HR = 1.093), AC073529.1, C9orf147, AC000068.1, and RPP38-DT (*p* < 0.1), were analysed and identified in combination with the clinical survival data. Figure 3B shows the expression of target lncRNAs in tumour samples and normal samples lncRNA box plots (Fig. 3C) and heatmaps (Fig. 3D) were obtained by the R packages "pheatmap", "reshape2" and "ggpubr". The high-risk lncRNAs associated with the prognosis are indicated in red, and the low-risk lncRNAs are indicated in green.

### Consensus Clustering Identified Two Clusters of CESC

The CESC cohort was classified into different clusters based on the expression of prognosis-related lncRNAs. When the cluster index "k" was increased from 2 to 9, k = 2 proved to be the best point to obtain the maximum difference between clusters and produced the least interference between clusters at this time. Then, the CESC cohort was divided into Cluster 1 and Cluster 2, where Cluster 1 contained 252 samples and Cluster 2 contained 52 samples. Cluster 2 represents the higher lncRNA score. However, no significant survival difference was found between the two groups by Kaplan–Meier survival analysis (*p* = 0.066).

### Clinical features between the clusters

**Fig. 4:**
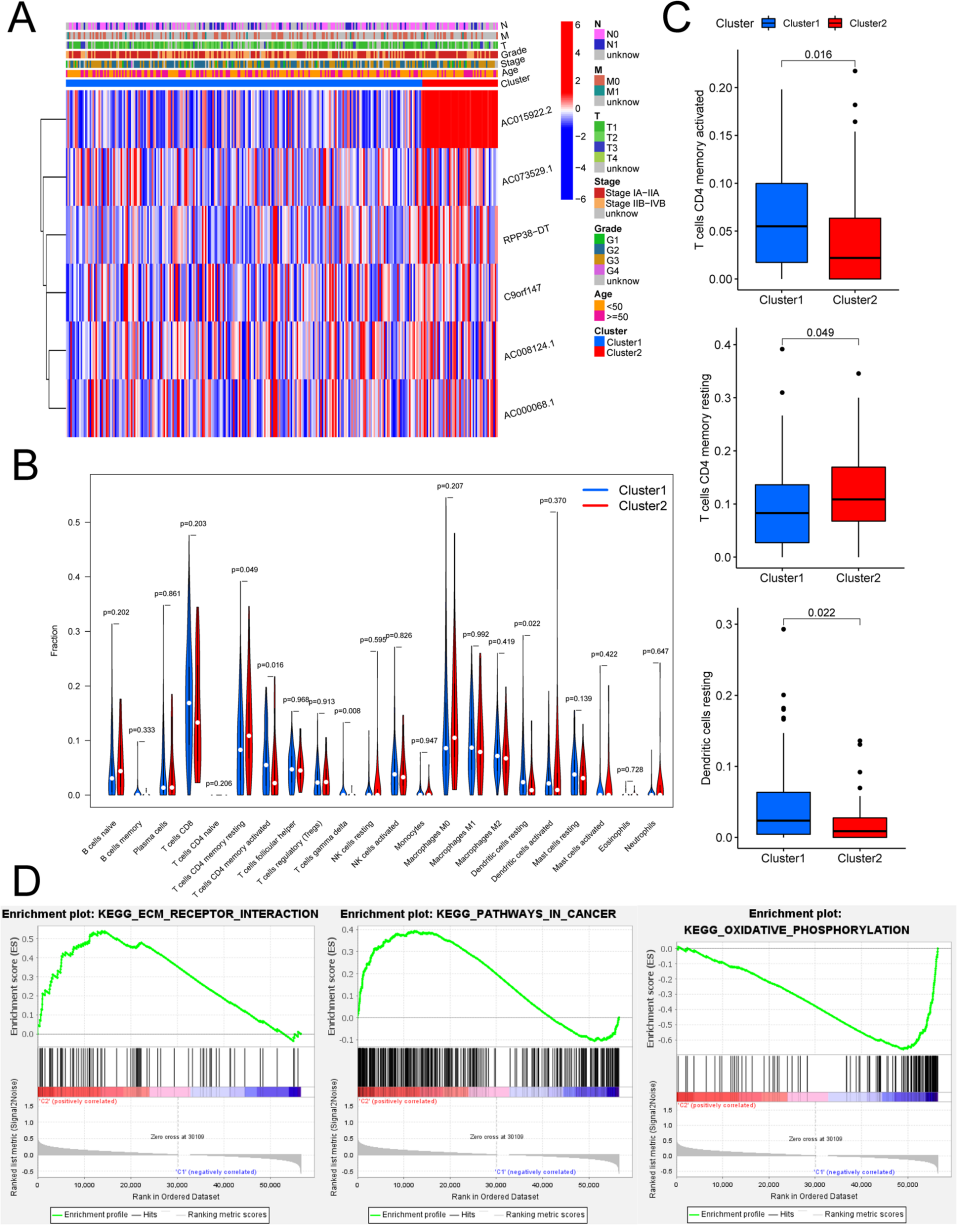
**A** Clinical features (including TNM staging, early (IA-IIA) and late (IIB-IVB) FIGO staging, histological grading, age > 50 years/ < 50 years, and clusters 1/2). Analysis of immune cell infiltration in CESCs. **B** Violin plots of immune cell differences between clusters 1/2. **C** Box plots of immune cell differences between clusters 1/2, where blue represents cluster 1 and red represents cluster 2. **D** Results of CESC gene set enrichment analysis (GSEA)

Then, the correlation between the two clusters and the clinical characteristics was analysed, as shown in Fig. 4A. We explored the relationship between the six lncRNAs mentioned above and TNM stage, FIGO (Federation International of Gynaecology and Obstetrics), stage, age, and grading, but the results showed that the correlations were not significant (*p* > 0.05).

### Analysis of immune cell infiltration in CESC

The R package "CIBERSORT" was used to obtain the results of the immune cell content in the CESC samples and to score the stromal cells and immune cells in the samples separately. The total score uses the combined score, i.e., the CIBERSORT score. Violin plots (Fig. 4B) and box plots (Fig. 4C) of the immune cell differences between the clusters were plotted using the R packages "vioplot" and "ggpubr". Differential analysis of immune cells between clusters showed that activated CD4 memory T cells (*p* = 0.016) and resting dendritic cells (*p* = 0.022) were highly expressed in Cluster 1 compared to Cluster 2, and resting CD4 memory T cells (*p* = 0.049) were highly expressed in Cluster 2 compared to Cluster 1. However, the scoring of the tumour microenvironment between the two clusters was not statistically significant.

### Results of the CESC tumour microenvironment enrichment analysis

Next, considering the strong association between the m^6^A-associated lncRNA scores and the prognostic and clinical features, we identified the genes and signalling pathways associated with m^6^A-related lncRNAs that influence clinical outcomes. Using the KEGG (Kyoto Encyclopedia of Genes and Genomes) database, we applied GSEA to examine the enriched gene sets that were obtained for Cluster 1 and Cluster 2 (Fig. 4D). The ECM receptor interaction (NES (normalized enrichment score) = 1.67, nominal *p* = 0.03), pathways in cancer (NES = 1.61, nominal *p* = 0.006), and other biological processes were enriched in Cluster 2, while oxidative phosphorylation and other biological processes were enriched in Cluster 1. Some of these gene sets were previously identified as being related to m^6^A modification. These results may provide some insight into the biological effects of m^6^A-related lncRNAs.

### Development of a Prognostic Signature

**Fig. 5:**
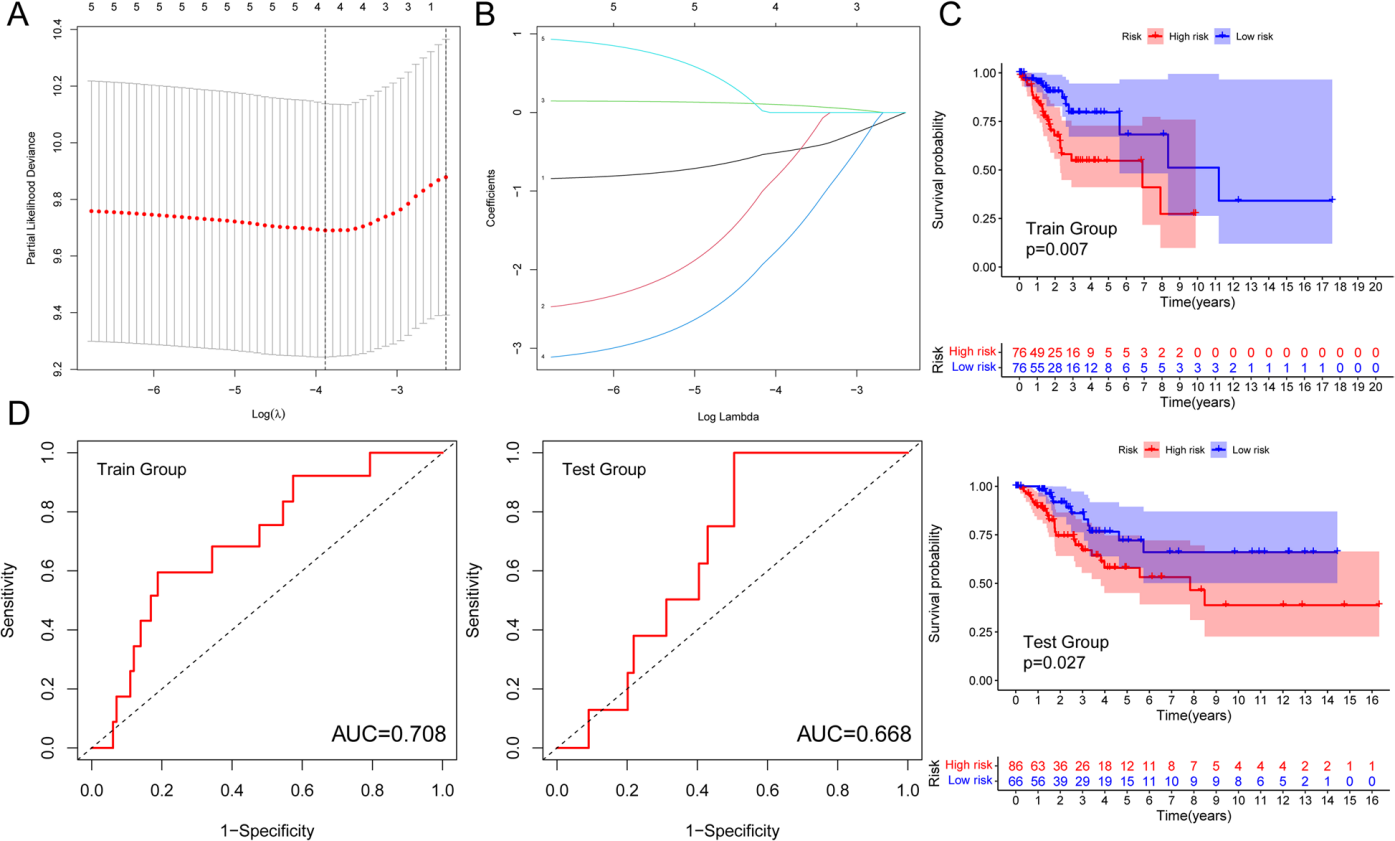
Development of a Prognostic Signature. **A** and **B** Least absolute shrinkage and selection operator (LASSO) regression was performed, calculating the minimum criteria. **C** Kaplan–Meier survival analysis for the training and testing groups. **D** ROC (receiver operating characteristic) curves were used to evaluate the prediction efficiency of the prognostic signature

A prognostic signature, including AC008124.1, RPP38-DT, AC015922.2, and AC073529.1, was developed using the LASSO Cox regression model according to the minimum criterion (Fig. 5A, B). The coefficients of AC008124.1, RPP38-DT, AC015922.2 and AC073529.1 were -0.4945, -0.7024, 0.0962 and -1.6514, respectively. The risk score for each CESC patient was therefore calculated with the following formula

$$\mathrm{riskScore}=\sum ({\mathrm{Coef}}_{\mathrm{i}}*1{\mathrm{ncRNA}}_{\mathrm{i}})$$

### where i is the expression of m^6^A-related lncRNA

**Fig. 6:**
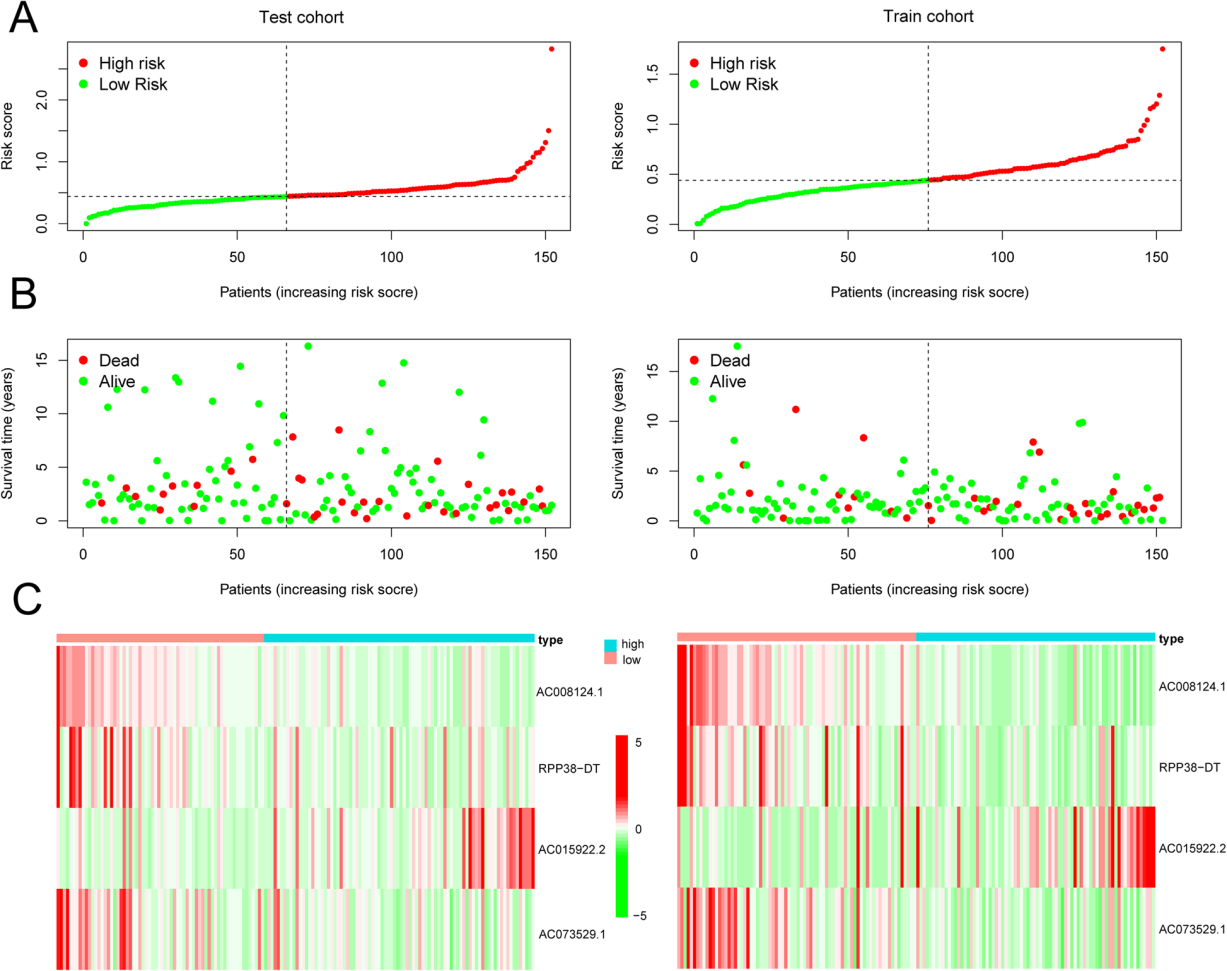
**A** Distributions of risk scores (red means high-risk score, green means high-risk score), **B** survival status (red means dead patients, green means alive patients) and **C** risk heatmap (red represents high expression, green represents low expression) of CESC patients based on the m^6^A-related lncRNA prognostic signature

To validate the prognostic value of this model, we divided the training (*n* = 152) and testing (*n* = 152) cohorts into high- and low-risk groups based on significant differences in OS determined by Kaplan–Meier curves (p_training_ < 0.01, p_testing_ < 0.05) (Fig. 5C). Based on the area under the curve (AUC) values, the model adequately predicted the OS rates for CESC patients in both cohorts (AUC_training_ = 0.708, AUC_testing_ = 0.668) (Fig. 5D). Risk profiles for the training and test groups showed that AC015922.2 was highly expressed in the high-risk group, while RPP38-DT, AC008124.1, and AC073529.1 were highly expressed in the low-risk group (Fig. 6).

### m^6^A risk scores as independent prognostic indicators

**Fig. 7:**
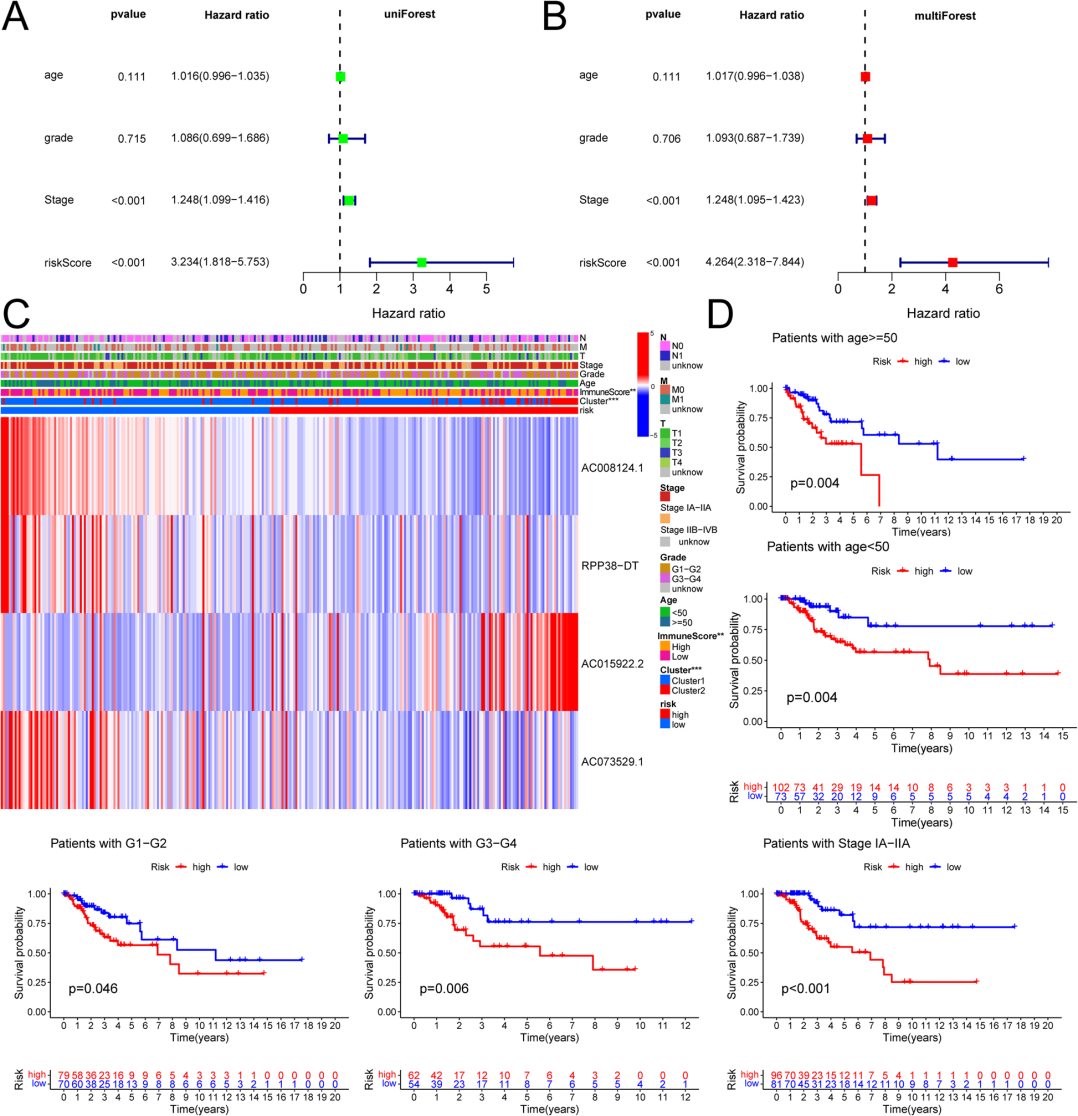
m^6^A risk scores as independent prognostic indicators. **A** Univariate Cox analysis of the clinicopathological features and risk score. **B** Multivariate Cox analysis identified the independent prognostic predictors. **C** The clinicopathological differences between the high- and low-risk groups. **D** Kaplan–Meier survival analysis of different clinical characteristics (patients age ≥ 50/ < 50, patients with G1-2/3–4, patients with stage IA-IIA) in the high-risk/low-risk groups

To further evaluate the prognostic value of the m^6^A-related lncRNA risk signature, factors including risk score, age, FIGO stage, and histological grade were successively included in the univariate and multivariate Cox regression models. Because the training and testing cohorts were derived from the same datasets and the sample size was limited, we subsequently merged all samples to increase the sample size. Univariate and multifactorial Cox regression analyses showed that the risk score and stage were significantly related to OS in both Cox analyses (*p* < 0.001) (Fig. 7A, B), indicating that the signature may be an independent prognostic tool.

### Association between m^6^A-related lncRNA risk scores and clinicopathological characteristics

Next, we evaluated the association between the risk scores and the clinicopathological features by producing a heatmap of the clinical characteristics, including TNM stage, histological grade, and FIGO stage, associated with the expression levels of the four selected regulators, where the immune score and cluster differed between patients in the high- and low-risk groups (Fig. 7C). No significant differences were detected among other clinical characteristics. Validation of the grouping by grading, staging, and age showed that the model we developed applied to different clinical groupings, including age < 50 (*p* = 0.04), age ≥ 50 (*p* = 0.004), stage IA-IIA (*p* < 0.001), G1-G2 (*p* = 0.046), and G2-G3 (*p* = 0.006). There were statistically significant differences in patient risk between age groups (age ≥ 50/age < 50, *p* = 0.047), immune scoring (high/low, *p* = 0.002), and clusters (Cluster 1/2, *p* = 1.3e*^−10^), and no statistically significant differences between patients with different stages and grades (Fig. 7D).

### Identification of m^6^A-related lncRNA risk scores associated with immune checkpoint molecules and immune cells

**Fig. 8:**
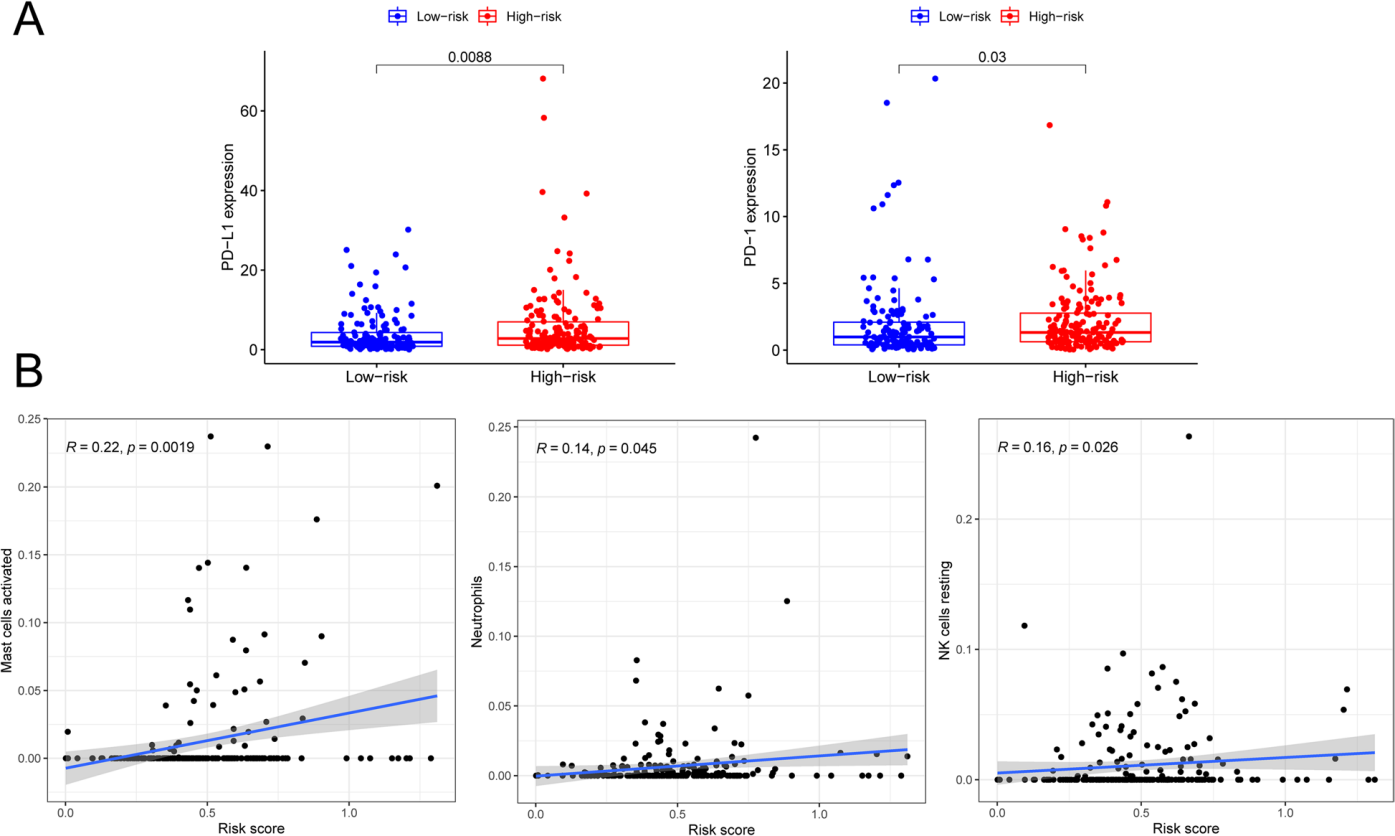
**A** m^6^A-related lncRNA modification of immune responses in CESC patients. **B** m^6^A-related immune cells in the tumour microenvironment in CESC patients

Next, we analysed the effects of m^6^A-related lncRNA modification on immune responses in CESC patients. The m^6^A-associated high-risk subgroup was associated with a significantly higher expression of several immune checkpoints, including programmed death 1 (PD-1) and programmed death ligand 1 (PD-L1), suggesting a potential response to anti-PD-1/L1 immunotherapy (Fig. 8A). For immune cells in the tumour microenvironment, activated mast cells (*p* = 0.002), neutrophils (*p* = 0.045) and quiescent NK cells (*p* = 0.026) were significantly activated in high-risk patients (Fig. 8B). It is suggested that immune cells in the TME may play a multifaceted role in the tumour microenvironment by mediating therapeutic resistance and immune tolerance in response to immune blockade. The mechanisms may be related to the regulation of various events in tumour biology, such as cell proliferation and survival, angiogenesis, aggressiveness and metastasis. In addition, it is possible that tumour-associated mast cells shape the tumour microenvironment by establishing crosstalk with other tumour-infiltrating cells [29]. Taken together, our work strongly suggests that m^6^A methylation modification patterns and m^6^A lncRNA-based risk typing are significantly associated with the response to PD-1/L1 immunotherapy and that the established m^6^A methylation modification profile will help predict the response to anti-PD1/L1 immunotherapy in cervical cancer patients. This finding needs to be further validated and confirmed in clinical practice [13].

## Discussion

As a reversible RNA modification process, m^6^A methylation has recently attracted much attention. However, how it plays a role in the development of cervical cancer in a lncRNA-dependent manner is still unknown [30, 31]. A growing body of research suggests that m^6^A modification plays an important role in the immune response, inflammation, and antitumour effects by interacting with different m^6^A regulators [32]. Although a large number of studies have revealed the epigenetic regulatory role of m^6^A regulators in the immune environment, the overall characterization of the m^6^A regulator-mediated TME is not fully understood [33, 34]. Therefore, identifying different m^6^A modification patterns in the tumour immune microenvironment will help provide insight into the interactions of m^6^A methylation in the antitumour immune response and help clinicians develop more precise tumour immunotherapy strategies [23, 35].

A total of 307 cervical cancer samples and three normal samples from the TCGA database were included in our study to explore the prognostic significance of the m^6^A-associated tumour microenvironment and lncRNAs. Four m^6^A-associated lncRNAs, AC008124.1, RPP38-DT, AC015922.2, and AC073529.1, were shown to have prognostic value in the TCGA dataset. These four lncRNAs have been reported to be associated with cancer progression; among them, Zhou et al. reported that lncRNA AC008124.1 regulated mRNAs in trans in breast cancer subtypes by competing for miRNAs [36]. Evans linked the upregulation of genes such as RPP38-DT to immunosuppressive therapy by gene enrichment analysis, suggesting that their interaction may be involved in the treatment of non-small-cell lung cancer [37]. Yang et al. identified AC015922.2 as a VHL (Von Hippel-Lindau)-associated lncRNA that is downregulated in ccRCC (clear cell renal cell carcinoma), whereas VHL gene inactivation is by far the most common oncogenic driver event in renal cell carcinoma [38].

Persistent infection of the cervical epithelium by human papillomavirus (HPV) and constitutive expression of viral oncogenes are thought to be the main causes of the complex molecular changes that lead to cervical epithelial cell transformation and cervical intraepithelial neoplasia [39]. Although lncRNAs AC008124.1, RPP38-DT, AC015922.2, and AC073529.1 have rarely been reported in HPV infection and cervical carcinogenesis development, we still speculate that the above lncRNAs may interact with chromatin modification complexes in specific regulatory regions to regulate gene transcription, and microRNAs (miRNAs) and circular RNAs (circRNAs) are jointly involved in the initiation and promotion of cervical cancer [39, 40]. Our future studies will also continue to focus on the up- or downregulation of target lncRNAs and observe their effects on important metabolic pathways in cervical cancer cells, such as STAT3, Wnt/β-catenin, PI3K/AKT and Notch, as well as high-risk HPV-encoded proteins, such as E6 and E7 oncoproteins.

We scored the CESC cohort patients according to their high or low expression of m^6^A-related lncRNAs and analysed the established independent prognostic model showing that patients with higher scores were usually accompanied by lower OS and worse clinical outcomes, a finding that was maintained in patients with cervical cancer of different grades, age > 50 years, age < 50 years and early stages. In the analysis of the tumour immune microenvironment, some studies point out that the TME shapes the fate of tumours by modulating the dynamic DNA (and RNA) methylation patterns of these immune cells to alter their differentiation into procancer (e.g., regulatory T cells) or anticancer (e.g., CD8 + T cells) cell types [41]. We found that high-risk subgroups were significantly associated with elevated levels of tumour-infiltrating lymphocytes and PD-L1 and PD-1, supporting the potential predictive value of immunotherapy.

The results of this study were derived and validated using the TCGA dataset for cervical cancer, but several limitations of our study remain. More independent cervical cancer cohorts should be used to validate the prognosis of m^6^A-associated lncRNAs. In addition, the role of lncRNAs and their interactions with m^6^A-related genes should be experimented with and confirmed using in vitro and in vivo approaches.

In summary, our study comprehensively evaluated the m^6^A modification patterns of 13 m^6^A regulators in 307 cervical cancer samples, established an independent prognostic model based on m^6^A-associated lncRNAs, and systematically correlated these modification patterns with TME cell infiltration characteristics. The above evidence suggests that m^6^A modifications are targeted to lncRNAs and that RNA methylation is important in the immune regulation of tumours. Assessing the m^6^A modification patterns of individual tumours will help improve our understanding of the infiltrative characteristics of the TME. We should pay more attention to the interaction and function of lncRNAs with m^6^A modifications to identify potential markers of prognosis and drugs for cervical cancer and refine therapeutic targets. Therefore, we hope that our findings will help identify prognostic lncRNAs that may be targeted by m^6^A modulators, thereby providing insight into their potential role in cervical cancer development, which can be applied in clinical practice to guide treatment options.

## Supplementary Information

**Additional file1:** **Figure S1:** Unsupervised clustering of the m6A regulators in the CESC cohort. **Figure S2:** Differential analysis of ICB-related genes among different clusters and normal/tumour samples. **Figure S3:** Differential analysis of lncRNA and ICB-related genes. **Figure S4:** Tumour microenvironment matrix score, immune score and total score between cluster 1 and cluster 2. **Figure S5:** Waterfall plot of tumour somatic mutations established by m6A and related lncRNAs. (produced by the maftools package)

**Additional file2:** **Table S1:** Each sample in the CESC cohort is divided into two clusters based on cluster analysis. **Table S2:** m6A and lncRNA genes derived from the CESC cohort. **Table S3:** Expression relationship between m6A and lncRNA in the CESC cohort. **Table S4:** Clinical characteristics of each sample in the CESC cohort. **Table S5:** Tumour microenvironmental characteristics of each sample in the CESC cohort. **Table S6:** Tumour microenvironment stromal score, immune score, and total tumour microenvironment score for each sample in the CESC cohort. **Table S7:** Construction of an independent prognostic model for the CESC cohort: factors and coefficients. **Table S8:** Survival time and survival status, lncRNA coefficient, risk score, and high/low risk classification for each sample in the CESC cohort.

## Data Availability

The datasets generated during and analysed during the current study are available in The Cancer Genome Atlas repository (https://portal.gdc.cancer. gov/). The source codes supporting the conclusions of this article are available on GitHub at https://github.com/zhanghe54321/m6acervival.git.

## Abbreviations

m^6^A: RNA N6-methyladenosine
TME: Tumour microenvironment
TCGA: The Cancer Genome Atlas
LASSO: Absolute shrinkage and choice of operator
TCGA: The Cancer Genome Cervical squamous cell carcinoma and endocervical adenocarcinoma
PD-1: Programmed death 1
PD-L1: Programmed death ligand 1
M5C: 5-Methylcytosine
M1A: N1-methyladenosine
ICB: Immune checkpoint blockade
CAF: Cancer associated fibroblast
BMDCs: Bone marrow-derived cells
DC: Dendritic cell
GEO: Gene-Expression Omnibus
GSEA: Gene-set enrichment analysis
ROC: Receiver operating characteristic
AUC: Area under the ROC curves
HR: Hazards ratio
OR: Odds ratio
Figo: Federation International of Gynaecology and Obstetrics
KEGG: Kyoto Encyclopedia of Genes and Genomes
