# Supplementary material for: N6-Methyladenosine-Related lncRNAs as potential biomarkers for predicting prognoses and immune responses in patients with cervical cancer
Source: BMC Genom Data. 2022 Jan 18;23:8. doi: 10.1186/s12863-022-01024-2 (PMC8767716; doi:10.1186/s12863-022-01024-2)

Figure S1. Unsupervised clustering of the m6A regulators in the CESC cohort.

A

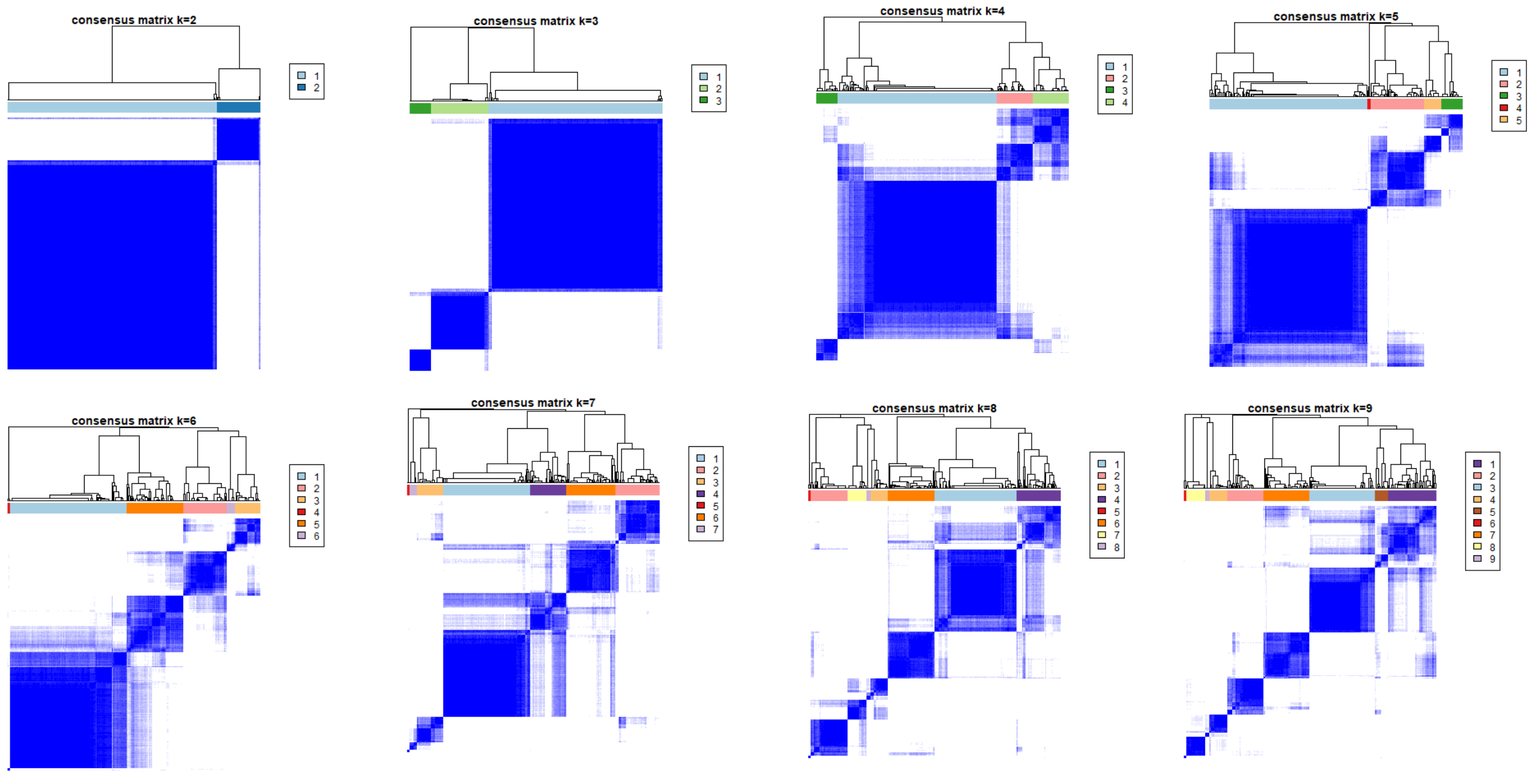

B

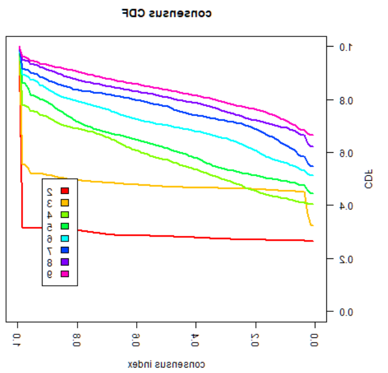

C

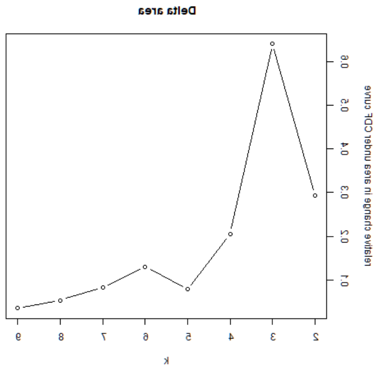

D

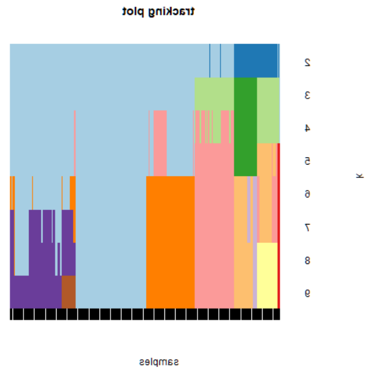

(A) The overlap between clusters when k between 2-9.
(B) Area under cumulative distribution function (CDF) curve when index k ranges from 2 to 9.
(C) Changes of length and slope of CDF curve when index k ranges from 2 to 9.
(D) Distribution of each sample in different clusters when k ranges from 2 to 9.

Figure S2. Differential analysis of ICB-related genes among different clusters and normal/tumour samples.

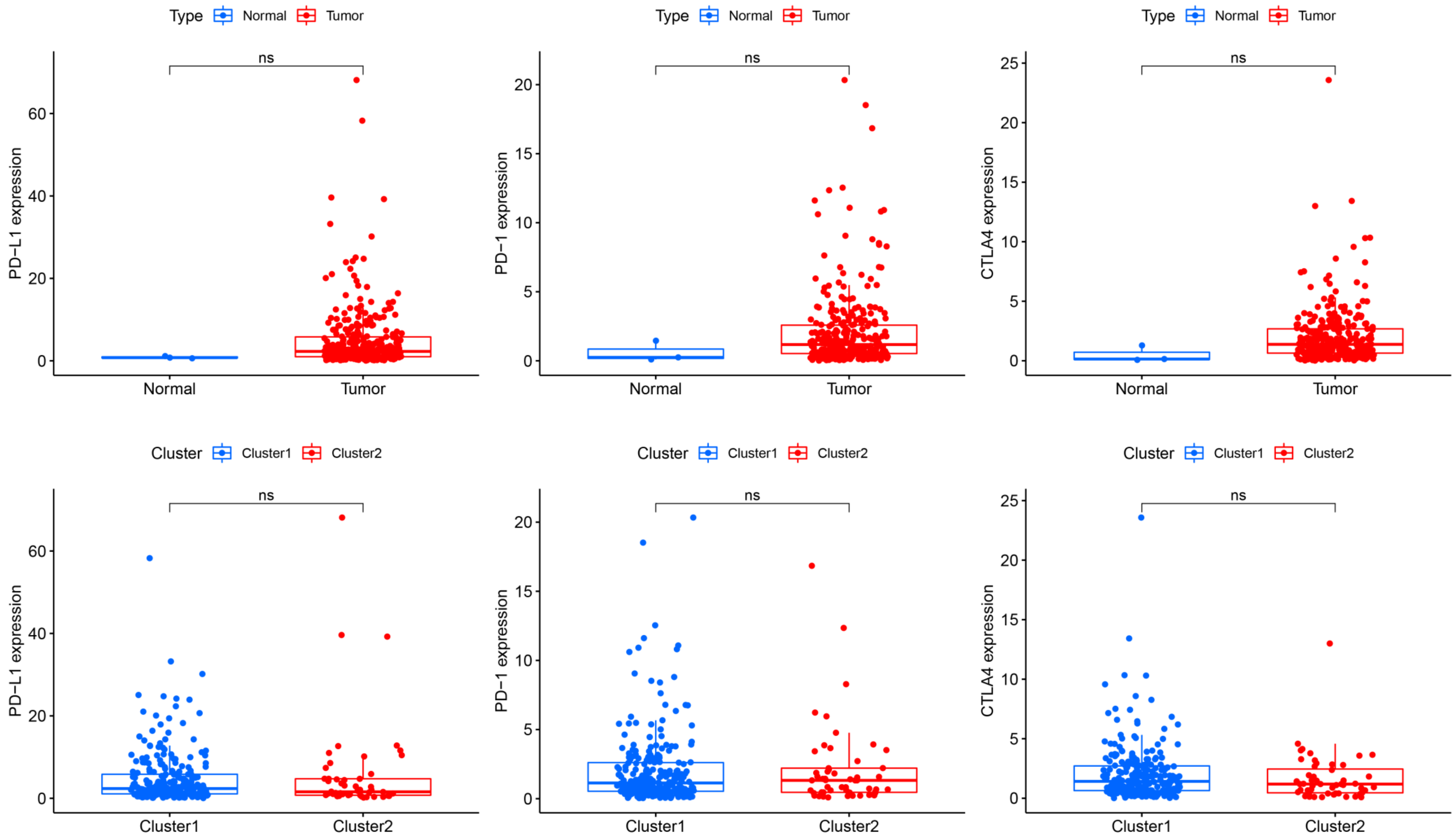

Figure S3. Differential analysis of lncRNA and ICB-related genes.

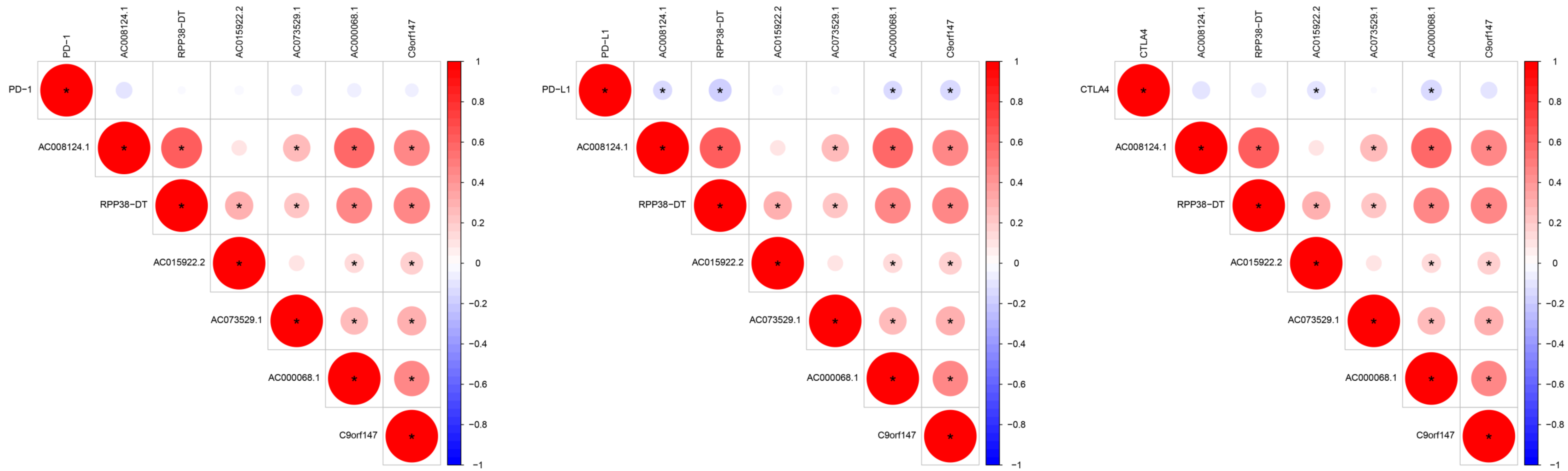

Figure S4. TTumour microenvironment matrix score, immune score and total score between cluster 1 and cluster 2.

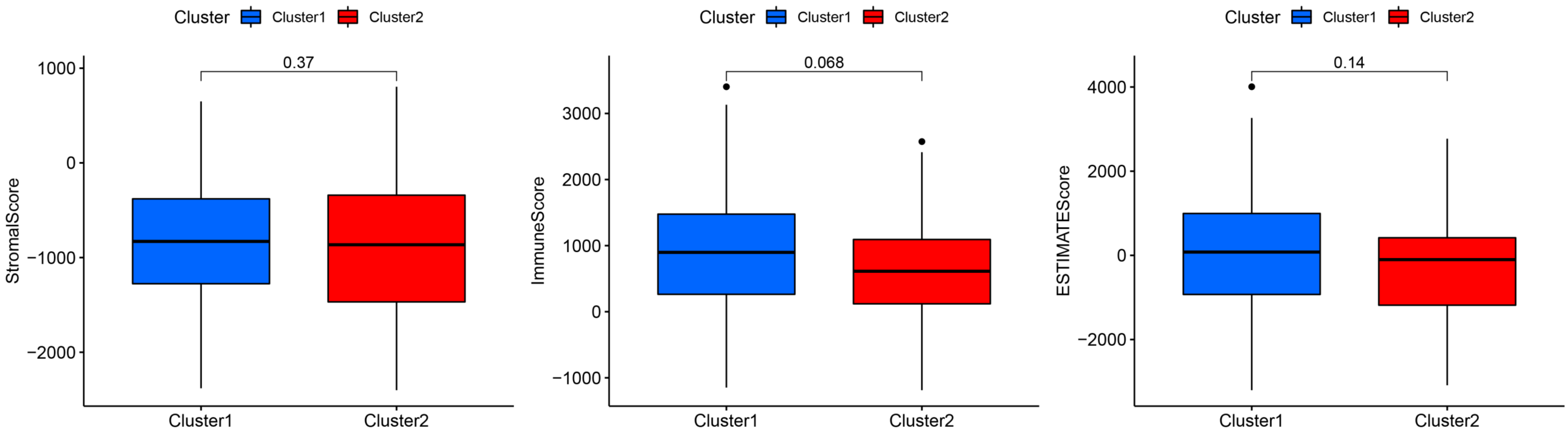

Figure S5. Waterfall plot of tumour somatic mutations established by m6A and related lncRNAs. (produced by the maftools package)

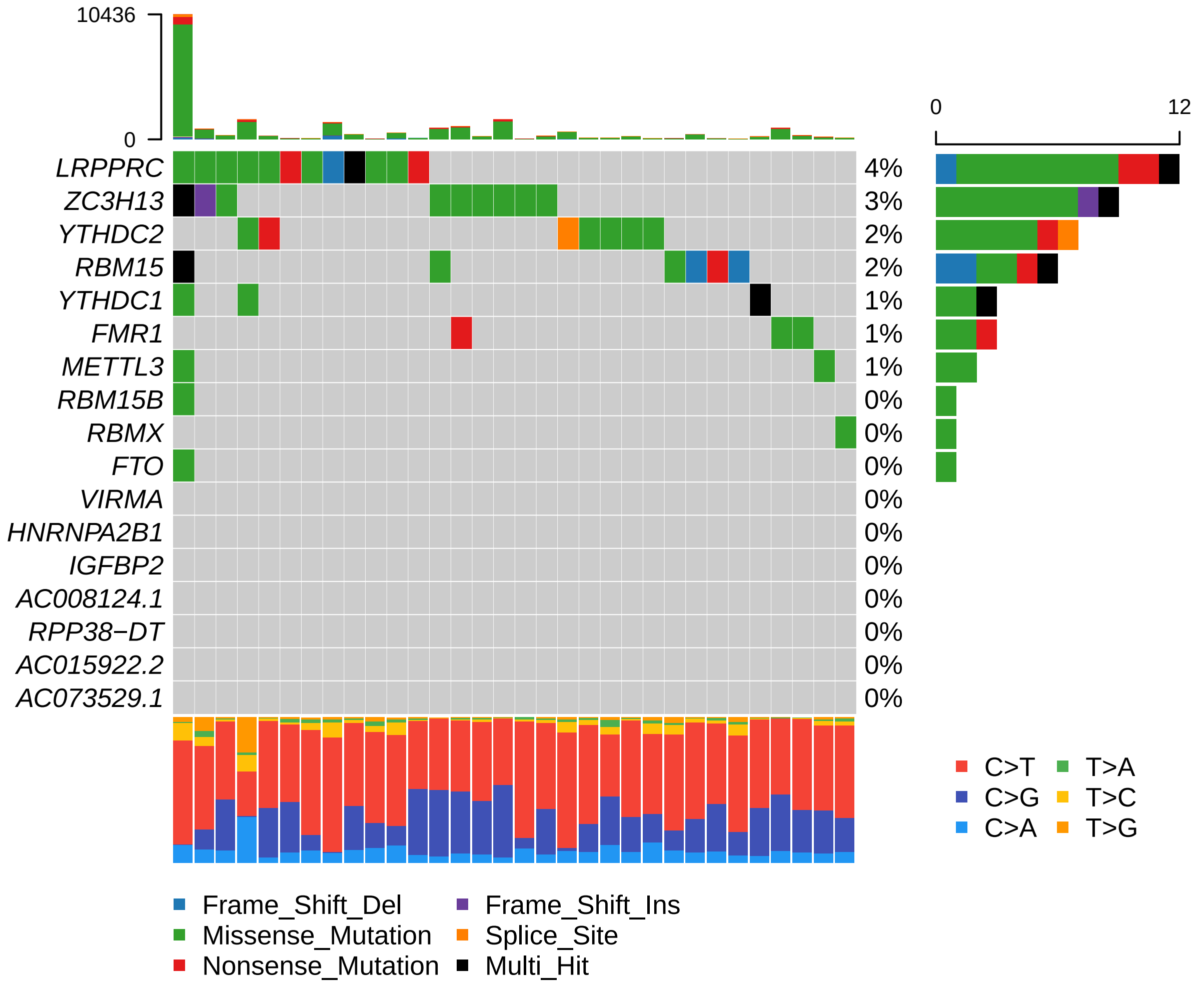

Supplement: Supplementary file 1 — Additional file1: Figure S1: Unsupervised clustering of the m6A regulators in the CESC cohort. Figure S2: Differential analysis of ICB-related genes among different clusters and normal/tumour samples. Figure S3: Differential analysis of lncRNA and ICB-related genes. Figure S4: Tumour microenvironment matrix score, immune score and total score between cluster 1 and cluster 2. Figure S5: Waterfall plot of tumour somatic mutations established by m6A and related lncRNAs. (produced by the maftools package) [file 12863_2022_1024_MOESM1_ESM.pdf]
